# Testosterone Enanthate: An In Vitro Study of the Effects Triggered in MG-63 Cells

**DOI:** 10.3390/biom12081159

**Published:** 2022-08-21

**Authors:** Benedetta Ghezzi, Ludovica Parisi, Elena Calciolari, Andrea Toffoli, Biagio Matera, Simone Lumetti, Giovanni Passeri, Guido Maria Macaluso

**Affiliations:** 1 Centro Universitario di Odontoiatria, Università di Parma, Via Gramsci 14, 43126 Parma, Italy; 2 Dipartimento di Medicina e Chirurgia, Università di Parma, Via Gramsci 14, 43126 Parma, Italy; 3 Laboratory for Oral Molecular Biology, Department of Orthodontics and Dentofacial Orthopedics, University of Bern, Freiburgstrasse 3, 3010 Bern, Switzerland; 4 Centre for Oral Clinical Research & Center for Oral Immunobiology and Regenerative Medicine, Institute of Dentistry, Barts and The London School of Medicine and Dentistry, Queen Mary University of London, London E1 4NS, UK; 5 Istituto dei Materiali per l’Elettronica e il Magnetismo-Consiglio Nazionale delle Ricerche (IMEM-CNR), Parco Area delle Scienze 37/A, 43124 Parma, Italy

**Keywords:** testosterone enanthate, osteoblasts, osteoinductive factor, human, tissue engineering

## Abstract

The aim of this study was to investigate the effects of the androgenic hormone testosterone enanthate (TE) on human MG-63 cells. MG-63 were cultured for 24 h in the presence of TE at increasing concentrations to assess its lethal dose. Therefore, the suitable concentration for a prolonged use of TE in vitro was assessed by viability assay over 9 days. Finally, MG-63 were exposed to TE for 14 days and assayed for differentiation by qPCR and Alizarin Red S staining. TE in the amount of 100 µM resulted as the maximum dose tolerated by MG-63 cells after 24 h. However, a prolonged exposure in culture TE in the amount of 100 µM showed a cytostatic effect on cell proliferation. On the contrary, TE 10 µM was tolerated by the cells and did not boost cell proliferation, but did enhance new bone formation, as revealed by *COL1A1*, *ALPL*, *BGLAP*, and *IBSP* gene expression after 3, 7, and 14 days, and calcium deposition by Alizarin Red S staining after 14 days. Based on the current study, 10 µM is the critical dose of TE that should be used in vitro to support bone differentiation of MG-63 cells.

## 1. Introduction

Reconstruction of bone defects in oral and maxillofacial surgery is often a major clinical challenge. Ridge remodelling following tooth loss is the most common cause for alveolar bone deficiencies in the horizontal and vertical dimension in the facial skeleton [1,2,3,4]. Additionally, more challenging defects are often the result of traumas, tumour resections, radiation-related osteonecrosis, and congenital anomalies such as lip and palate clefts [5,6,7]. To repair such defects, autologous bone (AB) graft is currently the gold standard [8,9]. Indeed, AB is the only clinically available material combining (i) viable osteogenic precursors (osteogenicity), (ii) a scaffold for new blood-vessel ingrowth and cell migration (osteoconductivity), and (iii) growth factors capable of triggering new bone formation (osteoinductivity) [10]. However, although AB transplantation is still considered the best option, large defects often require volumes of bone that are locally unavailable and that need to be harvested from a second surgical site, usually involving general anaesthesia, patient hospitalization, and significantly increased treatment costs [11]. Additionally, the morbidity associated with the second donor site, the unpredictable resorption rate of AB, and the risk of complications are major limiting factors. 

Tissue engineering represents a promising alternative option for bone regeneration. Indeed, by combining three building blocks, namely (i) autologous cells, (ii) a biomaterial scaffold, and (iii) bioactive molecules [12,13], it resembles osteogenic, osteoconductive and, osteoinductive properties, which are currently found only in AB grafts [14,15,16]. 

Different bioactive factors, including growth factors, enamel matrix derivatives, and autologous platelet concentrates have been proposed to enhance bone regeneration with heterogeneous evidence and are considered as good osteoinductive agents [17]. Among them, androgens (or androgenic hormones) are defined as natural or synthetic steroids which play a significant role in skeletal morphogenesis and the maintenance of bone homeostasis during the whole life [18]. A previous study by our group successfully tested their potential use as osteoinductive agents in a pre-clinical study in calvaria critical-size defects [19]. Testosterone enanthate (TE) is an analogue of testosterone, the most circulating androgen in men, which has shown positive effects on the regulation of bone turnover. The risk of side effects related to its systemic administration in women (i.e., extra hair growth, weight gain, and fluid retention) has limited not only its clinical application, but also in vitro study to investigate its efficacy as an osteoinductive agent. Nevertheless, the possibility of bioengineering TE with a biomaterial that would allow its local sustained and controlled delivery could potentially limit the side effects related to its potential release in the systemic circle [20].

Considering the aforementioned, the aim of the present study was to investigate the effects triggered by TE on bone-derived cells in vitro. Herein, we elucidate TE efficacy on proliferation, differentiation, and mineralization of human bone-like-derived cells, in order to identify a suitable dose for future pre-clinical studies. 

## 2. Materials and Methods

This study aimed to investigate the biological behaviour of MG-63 cells in the absence or in the presence of TE, in terms of proliferation, differentiation and mineralization. Our final goal was to assess the optimal androgenic concentration for the possible use of TE in the development of composite biomaterials for bone regeneration.

### 2.1. Cell Culture

Human osteoblasts MG-63 were obtained from the American Type Culture Collection (LGC, Standards s.r.l., Sesto S. Giovanni, Milano, Italy). Cells were cultured in Dulbecco’s Modified Eagle Medium (DMEM, Thermo Fisher Scientific, Carlsbad, CA, USA) supplemented with 10% heat inactivated Fetal Bovine Serum (FBS, Thermo Fisher Scientific, Carlsbad, CA, USA), 1% L-Glutamine (Thermo Fisher Scientific, Carlsbad, CA, USA), and 1% Penicillin and Streptomycin (PenStrep, Thermo Fisher Scientific, Carlsbad, CA, USA). Upon confluence, cells were trypsinized and seeded at the desired concentration for the in vitro experiments.

### 2.2. Cell Culture Treatments

Testosterone Enanthate (TE, ACME DRUGS s.r.l., Corte Tegge, Reggio Emilia, Italy) was added to cultured cells 24 h after seeding at concentrations ranging from 0.1 µM to 1000 µM.

### 2.3. TE Cytotoxicity

In order to evaluate the cytotoxicity of TE and its best concentration for further experiments, a Calcein-AM (Thermo Fisher Scientific) staining was performed 24 h after the addition of the steroid to the cells, which were seeded in 24-well plates at a concentration of 5 × 10^4^ cells/well. Cells were washed twice in Phosphate Buffer Saline (PBS, Sigma-Aldrich, Saint-Louis, CA, USA) and subsequently incubated with Calcein-AM at a final concentration of 4 µM in PBS for 15 min at room temperature (RT) in dark conditions. After extensive washing in PBS, specimen were mounted under glass cover slips with mounting medium (Dako Cytomation Fluorescence Mounting Medium, Agilent Technologies, Santa Clara, CA, USA) for photo bleaching reduction. Samples were observed with a fluorescence microscope Zeiss Axio Imager A.2 (Carl Zeiss, Jena, Germany) using a 10× objective. A semi-quantitative analysis of Calcein-AM positive cells was performed with the NIS-Element Br5.11 Software (Nikon, Tokyo, Japan) in order to quantify the number of live cells in every experimental condition.

### 2.4. Chemiluminescence

To further investigate the effects of the non-cytotoxic TE concentrations on cell viability, 5 × 10^3^ cells/well were seeded in triplicate in 96-well plates. Hence, the number of cells over time in the presence of TE was monitored by chemiluminescence using the CellTiter GLO assay (Promega, Madison, WI, USA). Cell viability was measured 1, 3, 5, 7, and 9 days after TE addition, following the manufacturer’s recommendations. In brief, culturing medium was removed, cells were rinsed twice in PBS and incubated with a solution of DMEM and CellTiterGLO Lysis buffer (1:1 dilution). Afterwards, samples were vigorously shacked for 2 min and the developed luminescence was stabilized for 10 min RT in dark conditions. Samples luminescence was finally assessed with a GLOMAX 20/20 luminometer (Promega, Madison, WI, USA).

### 2.5. In Vitro Osteogenesis

In order to evaluate the effects of the best TE concentration on bone differentiation, MG-63 were committed by osteogenic medium (alpha-Minimum Essential Medium (aMEM, Thermo Fisher Scientific, Carlsbad, CA, USA), supplemented with 10% FBS, 1% PenStrep, 0.05 mg/mL L-Ascorbic Acid (Sigma-Aldrich, Saint-Louis, CA, USA) and 0.01 M ß-Glycerophosphate (Sigma-Aldrich, Saint-Louis, CA, USA) with or without TE. Non-treated cells were used as a further control. Cells were seeded in their culturing medium at a density of 10^5^ cells/well of a 6-well-plate and were allowed to adhere to the culturing dish for 24 h under standard culturing conditions (37 °C ppCO_2_ 5%). Twenty-four hours after seeding, osteogenesis was induced. Cultures were kept for 14 days and the medium was exchanged every other day.

### 2.6. qPCR Analysis

Total RNA was extracted 3, 7, and 14 days after the addition of the stimuli to the cells and purified using the RNeasy^®^ Mini Kit (Qiagen, Hilden, Germany). RNA concentration and quality were assessed using a Nanodrop 2000c (Thermo Fisher Scientific, Carlsbad, CA, USA) and samples were stored at −80 °C until use. Next, 200 µg of RNA were used as the template for cDNA synthesis through the High-Capacity cDNA Reverse Transcription Kit (Applied Biosystems, Forster City, CA, USA). The analysis and quantification of the mRNA levels were performed by qPCR using Taqman Probe sets (Applied Biosystems, Forster City, CA, USA), a TaqMan Gene Expression Master Mix (both from Applied Biosystems, Forster City, CA, USA) on a StepOne Plus Real-Time PCR System (Applied Biosystem, Thermo Fisher Scientific, Carlsbad, CA, USA). The delta–delta Ct method was used to calculate relative mRNA expression levels and normalize values of each sample to *GAPDH*.

TaqMan Probes used: collagen type 1 alpha 1 (*COL1A1*, Hs00164004_m1), alkaline phosphatase (*ALPL*, Hs01029144_m1), bone gamma-carboxyglutamate protein (*BGLAP*, Hs01587814_g1), integrin binding sialoprotein (*IBSP*, Hs00173720_m1), and glyceraldehyde-3-phosphate dehydrogenase (*GAPDH*, Hs02758991_g1).

### 2.7. Mineralization Assay

Alizarin Red S (Sigma-Aldrich) was used in order to identify cultures’ mineralization 14 days after the induction of osteogenesis. At the end of the culturing time, cells were washed twice in PBS and fixed with 4% paraformaldehyde (Sigma-Aldrich) for 15 min at RT, thus rinsed twice in ddH_2_O and covered with a solution of Alizarin Red S 40 mM (pH 4.2) prepared in ddH_2_O. Cultures were subsequently incubated for 40 min at 4 °C on an orbital shaker. After extensive washing with ddH_2_O, specimen were air-dried before observation with an optical inverted microscope (Nikon).

After images acquisition Alizarin Red S concentration was measured by its solubilization. To this purpose, each well was treated with 400 μL of 10% acetic acid (Sigma-Aldrich) and incubated for 30 min at RT under shaking. Cells were then scraped from the plate, vortexed vigorously for 30 s, heated to 85 °C for 10 min, transferred on ice for 5 min and centrifuged at 20,000 rpm for 15 min at RT. After centrifugation, the supernatants were transferred into new eppendorf tubes, and the solution neutralized by adding 75 μL of 10% ammonium hydroxide. Sample absorbance was measured at 405 nm with a Multiskan^®^ FC microplate reader (Thermo Fisher Scientific, Carlsbad, CA, USA) and compared to a standard Alizarin Red S curve.

### 2.8. Statistical Analysis

Data were analyzed using Prism7 (GraphPad, La Jolla, CA, USA) and are reported as the mean ± SD. Differences between groups were evaluated with a two-way ANOVA statistical test and with a Tukey multiple comparison post-hoc test. Differences were considered significant when *p* < 0.05.

All the experiments were performed three times in triplicate.

## 3. Results

### 3.1. MG-63 Cells Can Be Maintained under Standard Culturing Conditions with the Addition of 10 µM of TE

**Figure 1:**
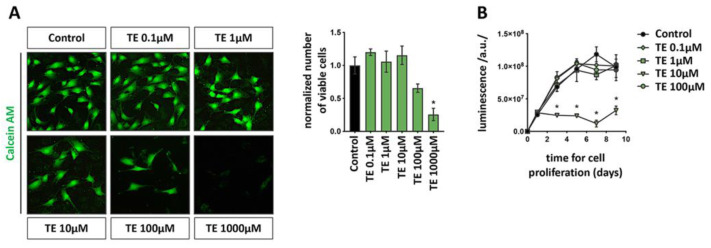
Identification of the TE dose to administer to MG-63 cells for prolonged in vitro culture. (**A**) Live imaging of MG-63 exposed for 24 h at the indicated concentrations of TE and incubated with Calcein-AM to detect viable cells (green). Scale bar: 50 µm. Number of viable cells is reported in the histogram to the right. Data are expressed as mean ± SD. *n* = 3. * = *p* < 0.05 vs. Control. (**B**) Cell viability curve of MG-63 cells incubated with increasing doses of TE for 9 days. Data are expressed as mean ± SD. *n* = 3. * = *p* < 0.05 vs. Control.

Our previous findings suggested that anabolizing hormones could be used in culture to promote bone differentiation of Saos2 cells up to a concentration of 1000 nM [21]. To extend these results, we tested the cytotoxicity of TE on a logarithmic scale range extending from 0.1 till 1000 µM. Calcein-AM staining (Figure 1A) confirmed that when administered at the maximum dose (1000 µM), TE resulted in being cytotoxic 24 h after administration (*p* = 0.0005). However, up to 100 µM, no evident signs of cytotoxicity were observed. Indeed, in all the tested groups, MG-63 osteoblasts adhered to the culturing substrate and presented with a spindle-like shape typical of these cell line. This observation prompted us to study the effect of TE concentrations up to 100 µM on cell viability over a period of 9 days (Figure 1B). When low TE doses (0.1, 1, and 10 µM) were administered to MG-63, the growth pattern perfectly fitted with the typical MG-63 growth curve (Control). The growth plateau was reached by all the tested groups 5 days after the addition of TE to the cultures, and no significant differences were observed among the Control and the treated groups. These results indicate that the addition of TE to the cultures does not produce any positive or negative effect on cell proliferation. On the contrary, a 100 µM dose of TE, which was not cytotoxic, resulted in being cytostatic and blocked the proliferation of the MG-63 in culture.

### 3.2. The Addition of TE in Culture Promotes MG-63 Differentiation

**Figure 2:**
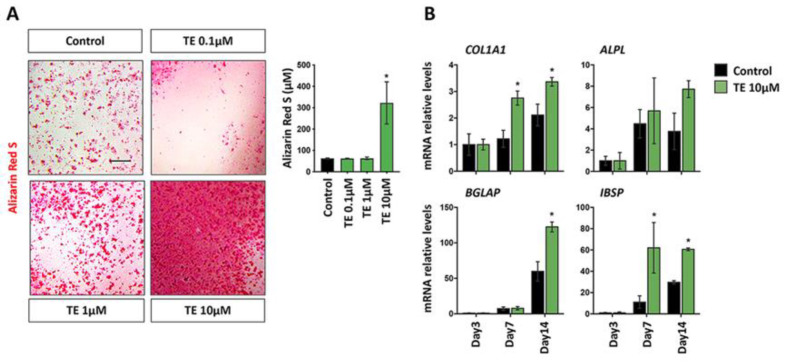
Effects of TE 10 µM on MG-63 differentiation. (**A**) Brightfield pictures of MG-63 cultures stained with Alizarin Red S after 14 days of differentiation with or without TE. Scale bar: 100 µm. The quantification of Alizarin Red S staining after its solubilization is reported in the histogram to the right. Data are expressed as mean ± SD. n = 3. * = *p* < 0.05 Control vs. TE. (**B**) qPCR data for the expression of the osteogenic-related genes *COL1A1*, *ALPL*, *BGLAP*, and *IBSP* during osteogenic differentiation with (green bars) or without TE 10 µM (black bars) after 3, 7, and 14 days of culture. Data are expressed as mean ± SD. n = 3. * = *p* < 0.05.

Since anabolic hormones are master regulators of bone differentiation [22], we assessed the differentiation potential of MG-63 in the absence or in the presence of TE. As a next step, we investigated the capacity of TE to induce bone differentiation. Alizarin Red S (Figure 2A) revealed that while TE administered at 10 µM induced a significant increase in culture mineralization compared to Control (*p* = 0.0288), while lower doses did not produce any effect on culture mineralization (*p* > 0.05). Therefore, we decided to investigate the expression of osteogenic differentiation markers such as *COL1A1*, *ALPL*, *BGLAP*, and *IBSP* with or without TE administered at the highest does (10 µM), which was shown to be the dose inducing a significant booster of the osteogenesis (Figure 2B). The early marker *COL1A1* was shown to be upregulated in the presence of TE already 7 days after the initiation of the commitment (Day 7 *p* = 0.0003; Day 14 *p* = 0.0038). Consistently, *ALPL* also showed a tendency to be increased in the TE group. However, no statistically significant differences were reported in this case. These data indicate an early effect of TE on the stimulation of bone differentiation, which was further enhanced on the long-term cultures. Indeed, late osteogenic markers such as *BGLAP* and *IBSP* were shown to be significatively increased after 14 days (*BGLAP* Day 14 *p* < 0.001; *IBSP* Day 14 *p* = 0.0013). 

## 4. Discussion

Effective constructs for bone tissue engineering require the combination of: (i) osteogenic cells capable of synthetizing and deposing new bone extracellular matrix; (ii) osteoconductive biomaterial scaffolds to support osteogenic precursor growth and differentiation, but also endothelial cells’ colonization and angiogenesis; and (iii) osteoinductive agents to stimulate osteogenic precursors, as well as tissue-resident mesenchymal stem cells [23]. In this regard, the use of androgens as osteoinductive molecules has been studied in the past and showed positive effects in preclinical studies [19,20]. Yet, their in vitro testing and use in clinical settings have been so far limited because of the potential side effects triggered by this kind of molecule when released into the systemic circle [24]. However, new advances in tissue engineering have paved the way for the possibility of immobilizing these molecules on the surface of biomaterials to guarantee their local activity. Additionally, as a consequence of these approaches, the amount of bioactive agent needed in situ would be much lower if compared to the amount that should be injected in the systemic circle to obtain the same local effect. TE is an androgenic hormone, analogue of testosterone, which we hypothesized as a good osteoinductive molecule for bone tissue engineering due to its influence on bone metabolism [22] and also in line with our preliminary findings [19,21]. We already showed that another androgenic hormone (stanozolol), played a pivotal role in the enhanced bone deposition if combined with DBBM granules, as well as it stimulated the increased mineral apposition rate and neo-vascularization [19]. However, the effects triggered by TE on osteogenic precursors have never been investigated so far. Since it is of outmost importance to clarify the potential effects and dose required to potentially support bone regeneration before embarking on a preclinical study, the present work investigated TE activity on human osteoblasts, with the aim of identifying an ideal concentration which should be immobilized on a biomaterial to locally test TE efficacy in vivo.

The current study showed that TE 10 µM does not affect the proliferation of human bone-like MG-63 cells (Figure 1) but possesses a strong potential to boost their differentiation (Figure 2). Remarkably, qPCR analysis showed a strong induction of *IBSP*, a gene involved in late osteoblasts’ differentiation, 7 days after commitment with a plateau stabilization until day 14, thus confirming the evident enhancement of bone differentiation in MG-63 cells when treated with the TE compared to the control group, as well as lower TE administered doses. Mineralization assay, with an evident stronger Alizarin Red S signal, further confirmed this observation. All these results are encouraging to support the hypothesis of using TE 10 µM for further in vivo pre-clinical experiments. Indeed, it can be speculated that TE would avoid undesired cell proliferation with consequent possible neoplastic stimulation, while promoting bone healing. In this regard, we are aware that being an osteosarcoma-derived cell line, MG-63 cells do not represent the best model for studying differentiation. However, in the past, substantial and relevant literature used this [25,26] or other similar models [21] to assess osteogenic properties of biomaterials and other substances, thus justifying their use also in this study. 

To the best of our knowledge, this is the first evidence in the literature that aims at investigating the effects triggered by TE on human osteoblastic-like cells. In our previous studies, we investigated the capacity of another androgenic hormone, stanozolol, to support bone-derived cells differentiation in vitro [21]. In that case, significantly lower doses of molecules were tested (range 1 to 1000 nM) and all were shown to promote bone differentiation. Hence, a 100 nM dose was chosen for further in vivo experiments. Such small amounts of stanozolol were adsorbed on deproteinized bovine bone mineral (DBBM) and were able to enhance early bone formation in a critical calvaria defect in rat (1 month), with no significant differences from the test group (DBBM only) in the long term (3 months) [19]. However, we also observed that in vivo stanozolol was completely released by DBBM within 24 h, challenging the possibility of loading higher doses and avoiding its undesirable release into the systemic circle and related side effects. In order to prolong the effects of local administration in vivo, loading concentration should be therefore increased. In the specific case of TE we observed that a dose of 10 µM (100 times more concentrated than the dose used for the stanozolol) was sufficient to significantly induce bone differentiation, without boosting or inhibiting the proliferation of MG-63 cells. We can therefore speculate that this dose when combined with an appropriate biomaterial scaffold could help to control its prolonged release and might be able to enhance bone healing in an in vivo model. Future studies are needed to test this hypothesis and explore the in vivo regenerative potential of TE.

## 5. Conclusions

Based on the obtained results, 10 µM seems to be the critical dose of TE to be used in vitro for the commitment of MG-63 osteoblastic cells and to support their bone differentiation.

## Data Availability

The datasets generate and/or analyzed during the current study are available from the corresponding author on reasonable request.
